# Self-Reported Symptoms and Pesticide Use among Farm Workers in Arusha, Northern Tanzania: A Cross Sectional Study

**DOI:** 10.3390/toxics5040024

**Published:** 2017-09-27

**Authors:** Wilbert Bunini Manyilizu, Robbinson Hammerton Mdegela, Arnfinn Helleve, Eystein Skjerve, Rudovick Kazwala, Hezron Nonga, Mette Hellen Bjorge Muller, Elisabeth Lie, Jan Lyche

**Affiliations:** 1 Health Systems Department, School of Public Administration and Management, Mzumbe University, P.O. Box 101, Morogoro 023, Tanzania; 2 Departmentt of Veterinary Medicine and Public Health, Sokoine University of Agriculture, P.O. Box 3021, Morogoro 023, Tanzania; rmdegela2012@gmail.com (R.H.M.); kazwala@gmail.com (R.K); nongahezron@yahoo.co.uk (H.N); 3 Institute of Health and Society, University of Oslo, International Community Health, P.O. Box 1130 Blindern, N-0318, 454 Oslo, Norway; Arnfinn.Helleve@medisin.uio.no; 4 Department of Food Safety and Infection Biology, Norwegian University of Life Sciences, P.O. Box 8146, 454 Oslo, Norway; eystein.skjerve@nmbu.no (E.S.); mette.hellen.bjorge.muller@nmbu.no (M.H.B.M); elisabeth.lie@niva.no (E.L); jan.l.lyche@nmbu.no (J.L.).

**Keywords:** pesticides, post-exposure effects

## Abstract

The objective of the study was to describe self-reported health symptoms, the use of personal protective gear and clothing and poor safety procedures when applying pesticides among farm workers. A total of 128 adult farm workers were interviewed using a structured questionnaire during the farming season. The commonly used pesticides included profenofos, mancozeb, chlorpyrifos, cypermethrin, deltamethrin, permethrin, lambda-cyhalothrin, endosulfan and carbosulfan. The majority (>90%) of farm workers used no personal protective clothing while handling pesticides. More than one-third of farm workers ate and drank without washing their hands following pesticide handling, while a smaller number smoked or chewed gum. Wearing special boots during pesticide application was found to reduce the risk of skin rash (OR = 0.2, 95% CI: 0.06–0.66), whereas smoking when applying pesticides increased the risk of chest pain occurrence (OR = 4.0, 95% CI: 1.14–15.43), as well as forgetfulness (OR = 4.0, 95% CI: 1.30–14.02). Chewing gum and eating when applying pesticides was associated with diarrhoea (OR = 11.0, 95% CI: 1.80–6.84 and OR = 7.0, 95% CI: 1.27–3.67 respectively). The increased self-reported prevalence of post-exposure adverse health effects among farm workers was associated with poor use of personal protective clothing and poor safety practices during pesticide use and handling. These data indicate the need for improved availability and use of protective equipment, and training in crop and pest management practices to prevent risky behavioursand for safer and sustainable vegetable production.

## 1. Introduction

Human health risks associated with pesticide exposure are a global concern [1]. The risk of adverse health effects is reported to be highest among agricultural workers in developing countries, as compared to developed countries. Developing countries use 20–30% of all agrochemicals produced worldwide [2] for improving and protecting crop yield. For example, in Tanzania, the imports of pesticides increased from 500 to 2500 tonnes between 2000 and 2003, and by 2006, a total of 300 different types of pesticides were registered and in use [3]. These includeendosulfan, which is restricted by the Stockholm Convention due to environmental persistence and toxic potential [4].

About a decade ago, safety measures were reported to be poorly implemented during pesticide storage, mixing, loading, spraying and disposal [5] in developing countries including Tanzania [6]. The unsafe use of pesticides has been reported to increase exposure levels, thereby increasing the risk of adverse health effects among exposed individuals [7]. Accordingly, in northern Tanzania, about 93% of farm workers are reported to have had acute pesticide poisoning incidences [8],withsymptoms such as headache, watering eyes, face and skin irritation, skin rashes, vomiting, diarrhoea, dizziness, and general body weakness [6,9,10]. The data addressing potential adverse effects of long-term exposure to moderate pesticide levels suggest a variety of adverse health conditions, such as central nervous-, reproductive- and immune system disorders, and cancers [2,4,11]. The effects of low, chronic exposure to pesticides have been largely reported in animals [11,12,13,14,15,16] but less so in humans [17,18,19]. Since symptoms related to exposure to pesticides in humans are not specific [20], a substantial underreporting of pesticideinduced health effects has been reported in Tanzania [21]. In addition, the Health Management Information System (HMIS) in Tanzania has only one category for all cases of poisoning, reflecting a lack of a comprehensive registry of adverse health effects associated with pesticide exposure. Thus, studies which provide scientific-based evidence documenting a link between unsafe pesticide-use and adverse health effects in Tanzania and other developing countries are strongly needed for the initiation of interventions and outreach to improve public health. In the study area, pesticides are applied to more than 5000 hectares of farmland for more than 270 days each year. The excess use of pesticides, coupled with inadequate personal protection, is likely to increase the exposure dose among sprayers [22]. Although the widespread use of pesticides has led to increased agricultural yields and protection of the harvests, their use has also resulted in increased health risks to workers as a result of poor protection, inappropriate use and handling, as well as living in close proximity to the pesticide application areas [23].

Even though the first reports on the extensive use and misuse of pesticides in Tanzania were published more than a decade ago, very few studies have assessed acute toxic effects, and no studies have investigated possible associations between long-term pesticide exposure and adverse health effects [24].

The objective of the present study was to describe current practices of pesticide use, including the use of personal protective clothing and equipment, and to establish possible associations between pesticide use and self-reported health conditions among farm workers.

## 2. Methods

### 2.1. Study Site and Participants

The study was conducted in 2015 among adult farm workers in two rural areas in northern Tanzania, namely Lake Eyasi and Ngarenanyuki in adjacent Karatu and Meru districts, respectively. The main crops produced for sale were tomatoes and onions in the Meru and Karatu districts, respectively, although the farm workers also produced smaller amounts of other crops both for sale and for their own consumption. A ward leader provided a person familiar to the farm workers who could act as an intermediary between researchers and the farm workers. One experienced and conversant (at least 5 years) farm worker, from a conveniently accessible farm plot cultivating onions and/or tomatoes, was selected for an interview and completion of a questionnaire formulated by the researchers. All farm workers were self-employed, working on their own small farm plotswithout supervision. One plot (one participant) out of 98 self-owned plots was selected. All participants were selected from an estimated 12,544 farm plots.

Using a cross-sectional study design, 128 farm workers were recruited for the study; 64 workers were from onion farms and 64 from tomato farms. Sample size of farm workers considered a 2-sided significance level of 0.05 and a power of 80% for equal sized samples.An assumption of p1 = 10% was made for self-reported disease (yes or no response) in those who reported personal protection and had not reported risky behaviours. An assumption of p2 = 30%was made for self-reported disease (yes or no response) for those who reported no personal protection but also practised any of the risky behaviours during pesticide handling. The two primary sampling populations had similar structure, size and farm worker behaviours, and it was assumed to provide equal weighting to the onion and tomato categories. All farm workers requested to join the study, consented, and were interviewed. No farm workers declined to participate.

### 2.2. Ethical Issues

Ethical approval to carry out this study was received from the Medical Research Coordinating Committee of the National Institute for Medical Research (Ref NIMR/HQ/R.8a/Vol. IX/1354), as well as to publish the results (Ref. NIMR/HQ/P.12 VOL XV/7). All participants were informed orally that participation in the study was voluntary and that the presentation of the findings would secure anonymity.

### 2.3. Data Collection 

Personal interviews using a structured questionnaire, lasting for 20 min, was conducted by investigators in farms where farm workers were at work. The questionnaire had been translated into English (then translated into local Swahili language) based on previous questionnaires used in the region [25,26,27]. The questionnaire included questions on pesticide handling behaviour and use. In particular, the questions focused on determining the reason for pesticide use and application procedure. Other questions included naming the pesticides the farm workers used and what the active ingredients, dose and application methods were. Additionally, they were asked about use of protective devices such as special hats, goggles, masks, overalls, gloves and boots in the last three months, as well as other activities such as smoking, eating, chewing gum and drinking when handling pesticides. The questionnaire also assessed adverse health effects by identifying the occurrence of 12 symptoms including persistent headaches, burning sensations in eyes/face, weakness, fever, watering eyes, skin rash, itching and skin irritation, dizziness, chest pain, forgetfulness, vomiting and/or diarrhoea after pesticide use.

The independent variables included personal protective devices such as gloves, goggles, mask, hat, overalls, and/or special boots used to protect the eyes, face, head, nose and mouth, trunk and legs. Risky practices and/or behaviours that are believed to increase hand-to-mouth contact when applying pesticides included smoking, eating, drinking and chewing gum. The questionnaire responses for both dependent and independent variables were categorized as no = 0, yes = 1.

### 2.4. Statistical Analysis

Statistical analyses were conducted using Stata (SE 11 for Windows, StataCorp LP, College Station, TX, USA). Two-way tables with measures of association using the Chi-square test were applied for bivariate analysis. After demonstrating the unadjusted influences, the same variables were included in a multivariable logistic regression model and tested using the ROC and the HosmerLemeshow test adjusting for site, gender, age, education level, farming district, and farm size. Other variables included in the model were wearing gloves, special boots, and overalls for personal protection, as well as smoking, eating, drinking and chewing gum during pesticide application. The dependent variables in the models were 12 disease symptoms including skin rash, headache, fevers, irritated and watering eyes, weakness, itching, dizziness, chest pains, forgetfulness, vomiting anddiarrhoea and face irritation. The emphasis of interpretation was based on adjusted odds ratios from the logistic models.

## 3. Results

### 3.1. Demographics

The mean age of respondents was 36.9 ± 10.7 years, ranging between 18 and 68 years. There was no significant difference (*p* = 0.1) in age between onion (Karatu) and tomato (Meru) farm workers. Farm workers worked for almost 8 h per day between 7.00 a.m. and 3.00 p.m. for six days of the week, in farms with a median size of 2 acres (min. 0 and max. 98). Other farm worker characteristics are shown in Table 1. Table 2 shows the distribution of crops for sale cultivated in the Karatu and Meru districts. The education level of about 90% of the study subjects was either limited to only primary education (seven years at school) or no formal education, meaning that some farm workers were unable to read and write in Swahili language. In addition, only a third (33%) of workers had a family size of 4 or less, while the remaining proportion of study subjects had families of at least 5. Only 23% of farm workers owned a farm between 3 and 5 acres, while the remaining farm workers owned plots of less than 3 acres.

### 3.2. Pesticide Use

The most frequently used pesticides mentioned by the 128 respondents included profenofos (74%), mancozeb (72%), endosulfan (36%), chlorpyrifos (31%), carbosulfan (30%) and pyrethroids (28%) including cypermethrin, deltamethrin, permethrin and lambda-cyhalothrin. Another commonly used pesticide was abamectin (19%). The main reasons for applying pesticides included killing insects and improving crop growth. Pesticides were also used for the purpose of protecting onions following harvest and allowing farm workers to wait for the best market price, although a similar procedure was not used for tomatoes. Table 3 shows the details and brand names of the most commonly used pesticides. The brand names of insecticides used by Karatu farm workers were not limited to, but included Supercron, Selecron and Profecron for profenofos. Polytrin was the most available brand name for profenofos among Meru farm workers. Odeon and Milthane were known brand names for fungicides among Karatu farm workers, and mancozeb, Farmerzeb, Oshothane, but also Milthane, among Meru farm workers. Others under use that were sporadically mentioned included Tankopa (copper oxychloride), blue copper (copper sulphate), galigan (oxyfluorfen) and many more.

The onion–tomato cultivation continued for a total of 9 months with approximately 270 days of active agricultural activities in a year.For three months between January and March, the production of onions and tomatoes is not possible due to heavy rains and periodic flooding, as well as the increased incidence of diseases. Thus, during this rainy season, the farms are used as paddy fields. During the onion growing season, farm workers routinely spray, on average, 635 L of pesticides per acre, at least once every week, which involves spraying 8–12 times until onion harvest. Similarly, for tomatoes, farm workers routinely spray their crops up to 6 times until harvest, whereby the number of sprayings depends on both their own farming experience and visual observations of pest levels, which changes constantly as pests migrate between sprayed and unsprayed plots. The main sources of information on use of pesticides for farm workers were fellow farm workers (24%), own experience (19%) and agrochemical shops (15%) during purchasing, among others. Only 8% of farm workers were able to receive some information from the extension officer. More than 70% of the farm workers routinely mixed several pesticides, making a cocktail of pesticides with the aim of synergising the effects of differently acting pesticides, in order to eradicate non-responding pests. The farm workers claimed that single pesticide treatments were not effective at eliminating pests. The practice of using a cocktail of pesticides was generally undertaken without prior local scientific research.

### 3.3. Personal Protection and Risky Behaviours

The level of personal protection was low; the majority of questioned workers reported that they mixed and sprayed without any personal protective devices. Out of 128 farm workers, only an average of 16 (13%) reported having applied at least one of the 6 types of personal protective devices in the past three months. The personal protective equipment used by farm workers were made up of wool, e.g., hat for head, leather, and synthetic or cotton shoes for feet. Others were cotton clothes for the body and sun glasses for the eyes. An average of 34 (27%) reported having smoked, eaten, drunk and/or chewed gum during pesticide handling in the past three months (Table 4 shows the details).

### 3.4. Self-Reported Disease Symptoms

Every disease symptom out of 12 (symptoms) had occurred to an average of 51% (66/128) farm workers in the past three months. Table 4 details the frequencies of self-reported symptoms.

### 3.5. Personal Protection, Risky Behaviours and Self-Reported Disease Symptoms

Although lack of personal protection and risky behaviours were common among all farm workers, there were some differences reported between the two groups of farm workers. Despite the differences, associations between personal protections and risky behavioural practices and self-reported medical symptoms were demonstrated in all groups. Table 5 summarises logistic regression odds ratios (OR), including (starred) confidence limits that demonstrate significant associations. The logistic regression models show associations between background factors, including age, education level, farm size and exposure (personal protection and risky behaviours), with twelve different self-reported symptoms among farm workers. It was revealed that wearing special boots during pesticide application reduced the risk of developing skin rash. Smoking was associated with an increased incidence of chest pain, while eating and chewing gum during pesticide use was associated with increased levels of diarrhoea.

## 4. Discussion

The present study found significantly high frequencies of self-reported disease symptoms and poor use of protective equipment, as well as other unsafe practices when using pesticides. The disease symptoms included dizziness, chest pain, memory loss, vomiting, diarrhoea and fever. These symptoms were similarly reported in two different studies, conducted in Cambodia and Bolivia, that suggested exposure to acute toxic doses of pesticides [9,20]. In these studies, symptoms of acute intoxication (chest pain, excessive sweating, body tremors and shortness of breath, headache, dizziness, tiredness, blurred vision and vomiting) were reported to have been experienced by farm workers following the routine application of pesticides without proper personal protection. Unsafe practices increase the risk of pesticide exposure, thereby increasing the risk of clinical and subclinical adverse health effects [21]. In the present study, the wearing of gumboots was the most common means of personal protection reported among farm workers. Those who used gumboots had a risk reduction of 80% of developing skin rashes, while the risk of developing skin irritations was reduced by 84% if the workers wore protective gloves (Table 5). The other personal protection devices for the trunk, hands, mouth, nose, face and head were rarely worn and reported. Both smoking and chewing gum may increase the pesticide exposure because of more frequent hand to mouth contact. The present study revealed that smoking during pesticide application significantly increased the risk of chest pain (OR_adj_ = 4.20) and forgetfulness (OR_adj_ = 4.27), compared to non-smoking individuals. Furthermore, chewing gum (OR_adj_ = 10.90) and eating (OR_adj_ = 7.10) when applying pesticides was found to significantly increase the risk of diarrhoea. Previous studies show that a lack of using personal protective gear is associated with increased risk of exposure, consequently leading to potential adverse health effects [22].

### 4.1. Pesticide Use, Personal Protection, Practice and Risk for Adverse Health Effects

Misuse and mishandling of pesticides with inadequate, or without, personal protective devices was documented in the present study, as well as elsewhere in Tanzania, suggesting a lack of effective safety protocols for protecting farm workers against pesticide exposure [8]. The number and kind of pesticides used is broad, and the specific pesticides used change from time to time, depending on prevailing requirements. This is influenced by previous crop yield experiences, as well as occasional local promotions [28]. Variation in handling practices and the use of pesticide mixtures underline the difficulties in achieving a precise measure of pesticide exposure among the farm workers. Moreover, despite all farm plots being in the same farmland, there is no system in place to harmonise pesticide spraying for proper pesticide and pest control. As a result, there are challenges relating to the control of pesticide use and accessibility.Farm workers are also challenged by limited awareness of how and what to mix, the number of pesticides to use per crop, the type and dose of pesticides to apply per square unit, and a shortage of technical extension officers [28]. It was considered that the average use in the present study is about two times higher than the reported use in the Phillipines, where the average pesticide exposure among vegetable farm workers in Benguet is 388.5 L per week [29].Associations between the unsafe handling and misuse of pesticides and improper use of personal protection, and increased risk for adverse health effects have been reported in other developing countries, such as Cambodia [9], Armenia [23], Uganda and Kenya [30,31] among others. Misuse and mishandling of pesticidesmay not only increase the exposure risk among farm workers but also to their family members [32], due to inadequate storage and improper disposal of pesticide containers [33]. In the present study areas, there were some pesticides in usewhich were not in the registered list provided by the regulatory authority, the Tanzanian Pesticide Regulatory Institute (2010). Such brand names included Verec, Enabo and others. These findings indicate that there was poor control of pesticide use and supervision, leading to the availability and use of unregistered pesticides. In addition, the majority of label instructions for pesticide handling and use were only given in the English language, which the majority of the interviewed farm workers could not understand. The exclusive use of English to illiterate farm workers is a major limitation, and may partly explain the pesticide misuse. Risky behaviours during the mixing and application of pesticides are common for both Karatu and Meru farm workers [34]. Ignorance of farm workers, especially those with only a small farm plot and large family, could partly be to blame for the continued improper handling and use of pesticides. Thus, malpractice and negative health consequences could persist, despite previous scientific reports, as farm workers were more likely to rely on advice given by non-professional personnel such as neighbours, among others.

The farm workers from both locations reported using mixtures of several pesticides in one tank, without knowing whether they mixed together pesticides with the same active ingredients. For example, when mixing togethertwo or three organophosphates (OPs), the concentration of the OP active ingredient will increase two to three times, thereby increasing the risk of inducing adverse health effects among the sprayers and others working in the field. Furthermore, the concentration of OP on the food will also increase, thereby increasing the risk of causing harmful effects to the consumers. Additional risk for increased exposure to the consumers comes from the practice of using pesticides for protecting onions post-harvest, for the purpose of waiting for the best market price.

Surprisingly, among the misused pesticides, endosulfan had a 36% prevalence of usage. Endosulfan presents a threat to both humans and the environment because of poor biodegradability and a long half-life. With an endosulfan ban initiated in 2012, the relatively high prevalence (36%) is substantial, and higher than other developing countries in Africa [8]. The occurrence of some persistent organic pollutants in using such endosulfan after the ban, however, suggests that the five-year exemption usage of endosulfan created a loophole for further misuse.

Previous studies in the late 1990s and early 2000s in the same areas reported that the high incidence of pesticide poisoning symptoms could be associated with the poor use of personal protective clothing and devices. These findings resulted in training projects for risk reduction [8]. However, the use of safety measures when using and handling pesticides today still appears to be as poor as it was 10–15 years ago.

The literature shows that safety challenges are common across many developing countries. For example, in Africa, the majority of farm workers have no pesticide-related training, neither do they use a full set of personal protective equipment, nor do they usually shower after work. In the present study, apart from wearing partial sets of personal protection, the findings demonstrate that farm workers protect themselves using inappropriate equipment that are made up of materials not resistant to harmful chemicals. Such materials include wool (hat), cotton or leather (shoes) and cotton clothes, which may play a role in enhancing the pesticide uptake instead of preventing it. These findings are in line with findings reported by Singh and Gupta [5] in India. The right personal protective materials recommended include chemical-resistant ones, not cotton, wool, or leather, and that they be applied for proper coverage of the body parts at risk of contact, ingestion and inhalation. This personal protective equipment includes complete long clothing (for skin), goggles (for eyes), long rubber gloves (for hands), respirators or masks (for noses), face cloths and water proof hats (for head), and gum (rubber) boots (for legs and foot) [29]. Additional challenges include pesticide stockpiling and poor disposal of empty pesticide containers in farming fields. There are also control loopholes, such asthe informal import of pesticides without the formal pesticide registration process [35]. Furthermore, limited knowledge among farm workers, poor use of personal protective equipment and poor hygiene all affect human safety. A study conducted in Uganda reports that increased risk of exposure is associated with a high prevalence of acute and chronic pesticide induced symptoms [31]. Surprisingly, Mokhele [36] in 2001 reported that despite awareness about the harmful effects of pesticide exposure, farm workers lacked adherence to safety regulations and use of sanitation facilities. Public safety in developing countries can also be explained by a number of challenges, including poor supervision, control and coordination of pesticide stakeholders, as well as failure of existing occupational health legislation to address the agricultural workplace adequately. Other challenges include lack of coordinated safety training and surveillance data [37]. In Tanzania, there is still a lack of efficient systems for the training, supervision and control of pesticide use. In addition, as for many other developing countries, Tanzania lacks pesticide quality control laboratories [38]. In order to improve safety practices for public health, the policies and regulations should be biologically and socioeconomically sound [39], along with the establishment of specific regions for international regulatory development, to facilitate the enactment of pesticide legislation in developing countries.

Addressing the local technical needs for crop production and protection during pesticide use is important. Better corrective options for safe use require further exploration, focusing on the need for appropriate systemic interventions regarding regulations and law enforcement. Furthermore, changes need to be implemented regarding the behaviour of farm workers when using pesticides. In particular, coordinated efforts must be made to adequately train workers in the safe handling and use of pesticides, including the use of protective equipment and improved personnel hygiene. Only these actions, together with increased dialogue between farm workers and the local distributors of pesticides, can lead to safer handling procedures and the subsequent reduction in pesticide related health problems. Additionally, translating instructions on labels into Swahili, the language that can be understood by the majority of local farm workers, is important.

### 4.2. Study Limitations

One of the limitations with the present study is the recruitment procedures. Recruitment of potential participants was coordinated via the ward leader/extension officer, which might increase the risk of selection bias. Due to infrastructural difficulties, irrigation water canals and river tributaries in the basins in the study area, means of transport were limited (bicycle, motorbike and on foot), and so a convenient sampling method was applied during recruitment. This sampling method has potential for introducing selection and information biases. During the data collection period, women (some above 60 years and under 18 years) were the majority gender group in the farms. According to an intermediary person, these women are not as conversant as men, and the majority are shy and not open to talking to newcomers. Only experienced and conversant adult men and women were recruited. This participant selection technique assisted by an intermediary familiar person could also introduce selection bias. In the search for eligible participants through all heavy pesticide using villages, it is considered that a representative population was satisfactorily achieved for reliable and valid data. The results of the present study could not be conclusive because they are based on a questionnaire and used cross sectional design estimates, in which exposure levels are not adequately measured.

## 5. Conclusions

The use and handling of pesticides with a lack of training, proper storage and disposal of pesticide containers, in addition to poor use of personal protective devices were documented in the present study, as well as elsewhere in Tanzania, suggesting a lack of effective safety protocols for protecting farm workers against pesticide exposure. The farm workers sprayed up to 12 times per growing season with no system in place to harmonise pesticide spraying. They also showed limited awareness on how and what to mix, the number of pesticides to use per crop, the type and dose of pesticides to apply per square unit, and a shortage of technical extension officers. This study also demonstrated a common practice of mixing several pesticides in one tank without knowing whether they mixed together pesticides with the same active ingredients. Another common practice was to spray the onions post-harvest to protect the crop during storage while waiting for a better market price.

The present study also demonstrated significant associations between high frequencies of self-reported disease symptoms, such as dizziness, chest pain, memory loss, vomiting, diarrhoea, and fever, and poor use of protective equipment and other unsafe practices. These results indicate the need for training in crop and pest management practices for safer and sustainable vegetable production in Tanzania and elsewhere.

The results of the present study allow future hypothesis formulation for the design of studies, which may enable the establishment of causal links between unsafe use of pesticides, and adverse health effects.

## Figures and Tables

**Table 1:** Socio-demographic backgrounds of study participants.

Variable	Category	KaratuFarm Workers (*n* = 64)	MeruFarm Workers (*n* = 64)	Total (*N* = 128)
Gender	Female	9 (14.1%)	0 (0%)	9 (7.0%)
	Male	55 (85.9%)	64 (100.0%)	119 (93.0%)
Age	18–29 years	21 (32.8%)	11 (17.2%)	32 (25.0%)
	30–39 years	24 (37.5%)	23 (35.9%)	47 (36.7%)
	40–49 years	14 (21.9%)	23 (35.9%)	37 (28.9%)
	50–59 years	3 (4.7%)	4 (6.3%)	7 (5.5%)
	60 years and older	2 (3.1%)	3 (4.7%)	5 (3.9%)
Educational level	Never to school	10 (15.6%)	3 (4.7%)	13 (10.2%)
	Primary school	49 (76.6%)	52 (81.3%)	101 (78.9%)
	Secondary school	5 (7.8%)	9 (14.1%)	14 (10.9%)
Household size	1–4 members	24 (37.5%)	19 (29.7%)	43 (33.6%)
	5–8 members	28 (43.8%)	41 (64.1%)	69 (53.9%)
	9 or more	12 (18.8%)	4 (6.3%)	16 (12.5%)
Farm size	0–1 acre	37 (57.8%)	15 (23.4%)	52 (40.6%)
	1.1–3 acres	16 (25.0%	31 (48.4%)	47 (36.7%)
	3.1 acres or more	11 (17.2%)	18 (28.1%)	29 (22.7%)

**Table 2:** Crops for sale and own use from the study participants’ farms in percentage.

Crop for Sale *	**Karatu Farm Workers (*n* = 64)**	**Meru Farm Workers (*n* = 64)**	**Total (*N* = 128)**
Tomato	5 (7.8%)	59 (92.2%)	64 (50.0%)
Onion	46 (71.9%)	17 (26.6%)	63 (49.2%)
Maize	25 (39.1%)	8 (12.5%)	33 (25.8%)
Beans	5 (7.8%)	3 (4.7%)	8 (6.3%)
Rice	8 (12.5%)	0 (0.0%)	8 (6.3%)
Green Pepper	2 (3.1%)	3 (4.7%)	5 (3.9%)
Other vegetables	6 (9.4%)	1 (1.6%)	7 (5.5%)
Crop for own use *	**Karatu**	**Meru**	**Total**
Tomato	1 (1.6%)	15 (23.4%)	16 (46.9%)
Onion	58 (90.6%)	35 (54.7%)	93 (72.7%)
Maize	3 (4.7%)	29 (45.3%)	32 (25.0%)
Beans	12 (18.8%)	19 (29.7%)	31 (24.2%)
Cabbage	6 (9.4%)	1 (1.6%)	7 (5.5%)
Other vegetables	2 (3.1%)	3 (4.7%)	5 (3.9%)

* All crops were sprayed with pesticides.

**Table 3:** Distribution of responses for some pesticides commonly used (brand names and active components) by crop farm type *N* = 128.

Active Ingredient	Brand Names	Karatu Farm Workers *n* = 64	Meru Farm Workers *n* = 64	Total *N* = 128
Abamectin	Balton 50EC, Abamectin 20EC	10 (7.8%)	14 (10.9%)	24 (18.8%)
Carbosulfan	Marshal 250C	37 (28.9%)	1(0.8%)	38 (29.7%)
Chlorpyrifos	Dursban 4E, Duduba 450EC, Twigafos 48EC	5 (3.9%)	35 (27.3%)	40 (31.3%)
Endosulfan	Thionex 35EC	46 (35.9%)	0 (0%)	46 (35.9%)
Lambdacyhalothrin plus others	Karate 5EC, Ninja 5EC and other pyrethroids	23 (18.0%)	12 (9.4%)	35 (27.4%)
Mancozeb	Milthane Super, Oshothane 80WP, Ridomil Gold 68WG, Victory 72WP, Ivory 72, Ebony M72, Odeon 720SC, farmerzeb (techn) etc.	40 (31.3%)	52 (40.6%)	92 (71.9%)
Profenofos	Profecron, Tanzacron 72E, Mocron, Selecron, Supercron, Mupacron 50EC	43 (33.6%)	52 (40.6%)	95 (74.2%)

**Table 4:** Self-reported health problems following use of protective gear and risky behaviours among farm workers applying pesticides.

Variables		Karatu Farm Workers (*n* = 64)	Meru Farm Workers (*n* = 64)	Total (*N* = 128)
Self-reported health	Burning sensation eye/face	40 (62.5%)	34 (53.1%)	74 (57.8%)
symptoms ever	Headache	46 (71.9%)	34 (53.1%)	80 (62.5%)
experienced after	Tiredness	44 (68.8%)	40 (62.5%)	84 (65.6%)
application of pesticides	Fever	54 (84.4%)	44 (68.8%)	98 (76.6%)
	Water eyes	38 (59.4%)	28 (43.8%)	66 (51.6%)
	Skin rash	46 (71.9%)	32 (50.0%)	78 (60.9%)
	Itching skin	48 (75.0%)	35 (54.7%)	83 (64.8%)
	Dizziness	44 (68.8%)	19 (29.7%)	63 (49.2%)
	Chest pain	41 (64.1%)	31 (48.4%)	72 (56.2%)
	Forgetfulness	26 (40.6%)	11 (17.2%)	37 (28.9%)
	Vomiting	21 (32.8%)	6 (9.4%)	27 (21.1%)
	Diarrhoea	19 (29.7%)	7 (10.9%)	26 (20.3%)
Personal protective devices used	Gloves	3 (4.7%)	8 (12.5%)	11 (8.6%)
during application of	Goggles	4 (6.2%)	0 (0.0%)	4 (3.1%)
pesticides during the last	Head protection	1 (1.6%)	1 (1.6%)	2 (1.6%)
three months	Mask	2 (3.1%)	2 (3.2%)	4 (3.1%)
	special boost	3 (4.7%)	42 (65.6%)	45 (35.2%)
	Overall	2 (3.1%)	33 (51.6%)	35 (27.3%)
Risky behaviour during	Smoking	22 (34.4%)	4 (6.2%)	26 (20.3%)
application of pesticides	Eating	13 (20.3%)	28 (43.8%)	41 (32.0%)
during the last three	Drinking	25 (39.1%)	25 (39.1%)	50 (39.1%)
Months	Chewing gum	15 (23.4%)	4 (6.2%)	19 (14.8%)

**Table 5:** Logistic regression of background and exposure practice factors associated with twelve different, self-reported symptoms among farm workers using pesticides *N* = 128.

		**Skin Rash**		**Headache**		**Fever**		**Watering Eyes**		**Itching**		**Weakness**
	**Adjusted OR**	**95% CI**	**Adjusted OR**	**95% CI**	**Adjusted OR**	**95% CI**	**Adjusted OR**	**95% CI**	**Adjusted OR**	**95% CI**	**Adjusted OR**	**95% CI**
**Adjusted for Variables**	**Model**		**Model**		**Model**		**Model**		**Model**		**Model**	
Age	1.01	0.97–1.06	0.99	0.96–1.03	0.99	0.95–1.05	1.01	0.98–1.06	1	0.96–1.05	1.01	0.97–1.05
Gloves	0.33	0.07–1.51	0.3	0.07–1.33	0.38	0.09–1.60	0.52	0.10–2.55	0.16 *	0.03–0.86	0.22 *	0.05–0.96
Special boots	0.20 *	0.06–0.66	1.1	0.37–3.03	0.49	0.14–1.68	0.56	0.18–1.71	0.6	0.19–1.89	0.79	0.26–2.38
Overall	1.09	0.34–3.48	2.4	0.79–7.12	0.43	0.13–1.47	0.65	0.21–2.05	0.66	0.21–2.07	1.68	0.53–5.29
Smoke	18.03 *	1.93–168.33	1.83	0.56–5.92	1.6	0.29–8.73	2.11	0.58–7.74	10.97 *	1.25–9.48	2.37	0.61–9.14
Eat	2.86	0.53–15.44	1.95	0.48–7.87	0.44	0.060–3.33	6.05 *	1.237–29.557	1.21	0.22–6.72	1.49	0.33–6.65
Drink	0.94	0.19–4.67	0.49	0.12–2.01	5.12	0.63–41.45	0.43	0.09–1.96	2.07	0.38–11.43	1.25	0.28–5.59
Chew gum	0.50	0.09– 2.84	2.72	0.66–11.25	0.83	0.12–5.99	5.49	0.93–32.50	0.42	0.07–2.47	0.86	0.19–3.90
	**Dizziness**		**Chest Pain**		**Forgetfulness**		**Vomiting**		**Diarrhoea**		**Face Irritation**	
	**Adjusted OR**	**95% CI**	**Adjusted OR**	**95% CI**	**Adjusted OR**	**95% CI**	**Adjusted OR**	**95% CI**	**Adjusted OR**	**95% CI**	**Adjusted OR**	**95% CI**
			**Model**		**Model**		**Model**		**Model**		**Model**	
Age	1.03	0.98–1.07	0.99	0.96–1.04	0.98	0.94–1.03	1.03	0.98–1.08	1.04	0.99–1.11	1.02	0.98–1.06
Gloves	0.23	0.02–2.19	2.5	0.55–11.14	-		-		-		0.29	0.07–1.28
Special boots	0.65	0.20–2.16	0.94	0.33–2.69	0.53	0.13–2.26	2.89	0.37–22.76	3.29	0.39–28.00	0.36	0.12–1.11
Overall	0.37	0.11–1.28	0.93	0.32–2.73	0.67	0.15–3.04	0.1	0.01–1.10	0.19	0.02–1.55	1.06	0.35–3.28
Smoke	3.17	0.82–12.28	4.20 *	1.14–15.43	4.27 *	1.30–14.02	3.46	0.95–.12.54	2.1	0.49–9.11	7.24 *	1.29–40.80
Eat	0.58	0.10–3.54	1.68	0.39–7.20	1.41	0.30–6.65	2.13	0.45–10.077	7.10 *	1.27–3.67	3.39	0.69–16.68
Drink	2.52	0.44–14.57	1.02	0.24–4.28	2.32	0.5–1.08	0.92	0.16–5.29	0.34	0.05–2.66	0.61	0.13–2.87
Chew gum	1.58	0.31–8.17	1.36	0.31–5.99	0.62	0.13–2.94	3.22	0.62–16.77	10.9 *	1.80–6.84	0.75	0.15–3.78

* OR was significant at *p* < 0.05.

## References

[1] Damalas C.A., Eleftherohorinos I.G. (2011). Pesticide exposure, safety issues, and risk assessment indicators. Int. J. Environ. Res. Public Health.

[2] Kesavachandran C.N., Fareed M., Pathak M.K., Bihari V., Mathur N., Srivastava A.K. (2009). Adverse health effects of pesticides in agrarian populations of developing countries. Rev. Environ. Contam. Toxicol..

[3] Lekei E.E., Ngowi A.V., London L. (2014). Pesticide retailers’ knowledge and handling practices in selected towns of Tanzania. Environ. Health.

[4] Stockholm Convention on Persistent Organic Pollutants. https://en.wikipedia.org/wiki/Stockholm_Convention_on_Persistent_Organic_Pollutants.

[5] Singh B., Gupta M.K. (2009). Pattern of use of personal protective equipments and measures during application of pesticides by agricultural workers in a rural area of Ahmednagar district, India. Indian J. Occup. Environ. Med..

[6] Ngowi A.V., Maeda D.N., Partanen T.J., Sanga M.P., Mbise G. (2001). Acute health effects of organophosphorus pesticides on Tanzanian small-scale coffee growers. J. Expo. Sci. Environ. Epidemiol..

[7] Koureas M., Tsakalof A., Tzatzarakis M., Vakonaki E., Tsatsakis A., Hadjichristodoulou C. (2014). Biomonitoring of organophosphate exposure of pesticide sprayers and comparison of exposure levels with other population groups in Thessaly (Greece). Occup. Environ. Med..

[8] Lekei E.E., Ngowi A.V., London L. (2014). Farmers’ knowledge, practices and injuries associated with pesticide exposure in rural farming villages in Tanzania. BMC Public Health.

[9] Jensen H.K., Konradsen F., Jors E., Petersen J.H., Dalsgaard A. (2011). Pesticide use and self-reported symptoms of acute pesticide poisoning among aquatic farmers in Phnom Penh, Cambodia. J. Toxicol..

[10] Kim J., Ko Y., Lee W.J. (2013). Depressive symptoms and severity of acute occupational pesticide poisoning among male farmers. J. Occup. Environ. Med..

[11] Alavanja M.C., Hoppin J.A., Kamel F. (2004). Health effects of chronic pesticide exposure: Cancer and neurotoxicity. Annu. Rev. Public Health.

[12] Sprince N.L., Lewis M.Q., Whitten P.S., Reynolds S.J., Zwerling C. (2000). Respiratory symptoms: Associations with pesticides, silos, and animal confinement in the Iowa Farm Family Health and Hazard Surveillance Project. Am. J. Ind. Med..

[13] Krimsky S., Simoncelli T. (2007). Testing pesticides in humans: Of mice and men divided by ten. J. Am. Med. Assoc..

[14] Wang F., Roberts S.M., Butfiloski E.J., Morel L., Sobel E.S. (2007). Acceleration of autoimmunity by organochlorine pesticides: A comparison of splenic B-cell effects of chlordecone and estradiol in (NZBxNZW)F1 mice. Toxicol. Sci..

[15] Gomes J., Lloyd O.L., Hong Z. (2008). Oral exposure of male and female mice to formulations of organophosphorous pesticides: Congenital malformations. Hum. Exp. Toxicol..

[16] Fukuyama T., Kosaka T., Tajima Y., Hayashi K., Shutoh Y., Harada T. (2011). Detection of thymocytes apoptosis in mice induced by organochlorine pesticides methoxychlor. Immunopharmacol. Immunotoxicol..

[17] Rosenstock L., Keifer M., Daniell W.E., McConnell R., Claypoole K. (1991). Chronic central nervous system effects of acute organophosphate pesticide intoxication. The Pesticide Health Effects Study Group. Lancet.

[18] McConnell R., Pacheco F., Wahlberg K., Klein W., Malespin O., Magnotti R., Akerblom M., Murray D. (1999). Subclinical health effects of environmental pesticide contamination in a developing country: Cholinesterase depression in children. Environ. Res..

[19] Colosio C., Alegakis A.K., Tsatsakis A.M. (2013). Emerging health issues from chronic pesticide exposure: Innovative methodologies and effects on molecular cell and tissue level. Toxicology.

[20] Jørs E., Morant R.C., Aguilar G.C., Huici O., Lander F., Bælum J., Konradsen F. (2006). Occupational pesticide intoxications among farmers in Bolivia: A cross-sectional study. Environ. Health.

[21] Lekei E., Ngowi A.V., London L. (2016). Undereporting of acute pesticide poisoning in Tanzania: Modelling results from two cross-sectional studies. Environ. Health.

[22] Prado-Lu L.D. (2007). Pesticide exposure, risk factors and health problems among cutflower farmers: A cross sectional study. J. Occup. Med. Toxicol..

[23] Tadevosyan A., Tadevosyan N., Kelly K., Gibbs S.G., Rautiainen R.H. (2013). Pesticide use practices in rural Armenia. J. Agromed..

[24] Aktar M.W., Sengupta D., Chowdhury A. (2009). Impact of pesticides use in agriculture: Their benefits and hazards. Interdiscip. Toxicol..

[25] Nonga H.E., Mdegela R.H., Lie E., Sandvik M., Skaare J.U. (2011). Assessment of farming practices and uses of agrochemicals in Lake Manyara basin, Tanzania. Afr. J. Agric. Res..

[26] Erbaugh J.M., Donnermeyer J., Kyamanywa S. (2010). Factors Associated with the Use of Pesticides in Uganda: Strategic Options for Targeting Integrated Pest Management (IPM) Programs.

[27] Hashemi S.M., Damalas C.A. (2012). Farmers’ perceptions of pesticide efficacy: Reflections on the importance of pest management practices adoption. J. Sustain. Agric..

[28] Manyilizu W.B., Mdegela R.H., Kazwala R., Müller M.B., Lyche J.L., Skjerve E. (2015). Self-reported health effects among short and long-term pesticide sprayers in Arusha, Northern Tanzania: A cross sectional study. Occup. Med. Health Aff..

[29] Lu J.L. (2009). Total pesticide exposure calculation among vegetable farmers in Benguet, Philippines. J. Environ. Public Health.

[30] Ibrahim M. (2015). Pesticides and health in vegetable production in Kenya. BioMed Res. Int..

[31] Oesterlund A.H., Sekimpi D.K., Maziina J., Racheal A., Jørs E. (2014). Pesticide knowledge, practice and attitude and how it affects the health of small-scale farmers in Uganda: A cross-sectional study. Afr. Health Sci..

[32] McCauley L.A., Anger W.K., Keifer M., Langley R., Robson M.G., Rohlman D. (2006). Studying health outcomes in farmworker populations exposed to pesticides. Environ. Health Perspect..

[33] Suratman S., Edwards J.W., Babina K. (2015). Organophosphate pesticides exposure among farmworkers: Pathways and risk of adverse health effects. Rev. Environ. Health.

[34] Blanco-Muñoz J., Lacasaña M. (2011). Practices in pesticide handling and the use of personal protective equipment in Mexican agricultural workers. J. Agromed..

[35] Negatu B., Kromhout H., Mekonnen Y., Vermeulen R. (2016). Use of chemical pesticides in Ethiopia: A cross-sectional comparative study on knowledge, attitude and practice of farmers and farmers in three farming systems. Ann. Occup. Hyg..

[36] Mokhele T.A. (2011). Potential health effects of pesticide use on farmworkers in Lesotho. S. Afr. J. Sci..

[37] Naidoo S., London L., Rother H.A., Burdorf A., Naidoo R.N., Kromhout H. (2010). Pesticide Safety Training and Practices in Women Working in Small-Scale Agriculture in South Africa.

[38] Matthews G., Zaim M., Yadav R.S., Soares A., Hii J., Ameneshewa B., Mnzava A., Dash A.P., Ejov M., Tan S.H. (2011). Status of legislation and regulatory control of public health pesticides in countries endemic with or at risk of major vector-borne diseases. Environ. Health Perspect..

[39] Schaerers G.A. (1996). Status of Pesticide Policy and Regulations in Developing Countries. J. Abtric. Entomol..

